# The Climatic Treatment of Disease

**Published:** 1886-01

**Authors:** Henry O. Marcy

**Affiliations:** Boston


TEXAS Medical ★ Journal,

PUBLISHED MONTHLY AT

AUSTIN, TEXAS.

Vol. 1.] JANUARY, 1886. [No. 7.

*"Scribimus indocti, doctique!"*

### Original Articles.

☞ CONTRIBUTED EXCLUSIVELY TO THIS JOURNAL. ☜

*The Articles in this Department are accepted, and published with the understanding that we are not responsible for, nor do we indorse the views and opinions of the writers, by so doing.*

### THE CLIMATIC TREATMENT OF DISEASE.

*By Henry O. Marcy, A. M., M. D., of Boston.*

Read before the American Academy of Medicine, New York, October 29, 1885.

For Daniel's Texas Medical Journal.

MODERN science invests old topics with new interest. The fundamental factors of sanitation are now so well understood that the question of climate and its influence upon health and disease may be discussed with the assurance of dealing somewhat, at least, with objective factors instead of theory and the results of experience alone. Every student of modern medicine finds a new inspiration from the great progress already made by the indefatigable labors of a considerable number of trained workers in all the leading countries. The one object of research has been to ascertain the causes of communicable diseases.

Few will now be found to question the real profit arising from this most difficult problem of scientific research, and that at least a clew has been obtained, leading to a knowledge of one of the most profound of nature's mysterious workings. There are many who accept it as the prophecy of a revelation to come, which shall elevate medicine from the domain of art, and place it within the realm of the more exact sciences; a knowledge of fundamental factors upon which to erect anew the Temple of Æsulapius.

If a contagium vivum plays the role in the entire group of zymotic diseases, the study of organic decomposition is not alone a question of chemical interest, but irresistably leads to conclusions of a biological character, and the scientist finds himself absorbed in watching life processes, a struggle for the survival of organisms with their environment, a conflict ending often in the destruction of the higher complex forms of animal life by a foe, so insignificant, so utterly beyond ordinary comprehension, that it seems verily a battle with the powers and principalities of the air.

The so-called "Germ Theory of Disease" has passed from the realm of theory into that of demonstration as fact, and based upon our present knowledge, confessedly fragmentary and imperfect, yet sufficiently exact from which to make deductions, sanitation becomes, in a large measure, a science. From this standpoint, climatology is invested with a new interest and value as seen especially in the contributions of the last few years. A surgical clean wound means an aseptic wound. The aphorism of Hippocrates "pure air, pure water, pure soil," signifies now an air, water and soil free from infectious elements of disease, and this must be defined as the most important factor pertaining to a health resort sought under the name of climate. This subject, which is far too large for the present occasion must be epitomised in a general way, as the individual wisely adapted to his surroundings.

Life carries with it, as an inherent property, a vital, resisting power by which the individual is able to adapt himself to surroundings and overcome deleterious influences. These phenomena we call the laws of life, beyond the limits of which life itself ceases. Thus heat, water, food, oxygen, etc., must be furnished within fixed limits. Departures from the ideal standard which we call health lessen the vital, resisting power of organs, as well as of individuals, and pave the way for the introduction of pathological factors.

Nature has provided man with an armor efficient to withstand and repel the invisible foes with which he is ordinarily surrounded. An unbroken skin is a coat of mail invulnerable to his surgical foes. The steady police superveillance of the cillia of the epithelium which line our respiratory passages are ever on watchful guard to seize and expel the intruder, be it bacillary or any other form of atmospheric contamination. When the defenders of the castle are disabled, then it especially behooves us to take into watchful consideration the strength and character of ourselves, as well as our enemies.

The chemical and mechanical laws of life have hitherto almost entirely engaged the attention of investigators in the study of the circumfusa of the invalid. For a number of years Committees from the American and British Medical Associations have been engaged in securing data for the comparison of the invasion of acute diseases with meteorlogical changes. Although these latter are largely factors in the problem, they have a much wider significance than usually supposed, since they make possible conditions not only devitalizing to the individual, but favoring in many instances the growth and development of infectious germs.

The tides and changes of the great atmospheric ocean in which we are submerged, are of the utmost significance. Its weight, as measured by the barometer, indicates the pressure which our bodies sustain. The moisture in suspension not only increases the weight, but modifies its influence upon the individual, and this is measured by the hygrometer. The changes of atmospheric temperature, as recorded by the thermometer, are of the utmost importance. The atmosphere also varies in its composition, of which, from our present standpoint of consideration, oxygen and ozone may be accepted as the most significant, since these agents materially lessen the growth and development of all the microscopic forms of plant life. From the observations of a number of our most competent physiologists, it is probable that the amount of oxygen absorbed in health does not vary greatly whether the individual is at the sea-level or at high altitudes. If this is correct, the accepted belief of increased oxygenation of the blood from breathing an air rich in oxygen, as at the sea-level, is not true.

The effect upon the individual of an ozonized atmosphere is generally conceded to be beneficial, yet, unfortunately, less is known upon this subject than could be desired; its antiseptic properties are well recognized and its influence upon the purity of the atmosphere, if not upon the individual, is undoubted. Ozone owes its great influence to its powerful oxidizing qualities. The compounds of ammonia, phosphorus, and sulphur are acted upon with great rapidity, and the odors resulting from animal decomposition are removed instantly. It is probably destructive to all the minute vegetable organisms when in active development, but its effect in destroying the vitality of their spores has not yet been ascertained.

This has its direct bearing upon the question of climate, since it has been pretty well determined that all the varieties of pathogenic microbes not only do not thrive, but fail to live, except within a narrow range of conditions.

Pasteur has placed his problem of protection from disease by the inoculation of attenuated virus of repeated cultures, upon the belief that these morbific bacteria have lost their specific effects in the presence of free oxygen—that is, these growths are anærobic. The experiments of Ogsten and Cheynne do not, however, confirm this supposition.

In a series of cultures of the vaccine coccus, conducted with great care, the cocci bred true and cultivated readily, but the vaccinations which I made from the cultures were entirely without effect. A few degrees in temperature will often be sufficient to retard, or destroy the development of the bacterial reproduction.

Based upon the belief that an aseptic atmosphere is of the first importance in the selection of a climate for the benefit of sufferers from a certain class of diseases, examinations of the atmosphere have been carefully made to demonstrate its purity. Miguel and Freudenrich found that microphytes were rare at eight hundred metres, and absolutely wanting at two thousand metres above sea-level. For physical reasons, high altitudes have been believed to be beneficial in early phthisis, and, influenced by the latter demonstrations of a bacillary cause, this aseptic state of the atmosphere is theoretically accepted as promising good results, and sanitary stations for mountain-air treatment of consumptives have been established at several selected points in the Alps, where the comforts of home life are furnished invalids. Of these health resorts Davos and St. Moritz are the most popular. St. Moritz is six thousand feet and Davos is over five thousand feet above the sea level, and the latter has become noted for winter treatment, although the average temperature from November to March is only 23 degrees F. The number of visitors last winter was over one thousand. The patients are urged to be active, out of doors within the limit of fatigue. Each year adds to the number of invalids resorting to these mountain sanitaria and the statistics show good results. It should, however, be borne in mind that the cases sent to these resorts are those selected in the incipient stages of disease, and this is to be taken into the account in the deductions that the recoveries average one-fourth; also the time elapsing after the treatment, has been too short to determine with accuracy the results. This pertains in criticism to the statistics of other similar localities, but it is by no means an easy task to secure satisfactory data and such a problem can never be subjected to a mathematical analysis.

A recent medical visitor writes as follows: "I saw the well-known "apostle" of Davos, Dr. Spengler, and his son-in-law, Dr. Peters, whose cordial reception I will not forget to mention here. They gave me much valuable information about Devos and its value as a health resort. They seem to aim chiefly to strengthening the general system, and the heart in particular. I do not think they make systematic use of the milk cure or of the douche; but the methodical walks up hill form part of the curative system. Consumptive patients benefit by their stay here if the heart be in good functional order, and if they have still a certain amount of strength. Erethic, or very weak patients sometimes lose their sleep as soon as they are here; and, unless they regain it after the first few days of acclimatisation they do better to leave the place altogether. Davos seems to be favorable also in the case of torpid, scrofulous patients, who want a bracing and exciting atmosphere, for non-irritable anæmics, and debilitated and convalescent subjects. The fear of tuberculous infection in these large sanitaria keep away a number of such patients, until special hotels have been opened for non-tuberculous persons. I spoke with our professional brethren on the occurrence of hæmoptysis, and was told that it was by no means more frequent here than in the plain. In a pamphlet, "Die Landschaft Davos," I find the following instructive data given by Dr. Spengler himself: 'Out of 323 patients 178 never had hæmoptysis, either at home or here, (in Davos); 126 had hæmoptysis at home and none here; 16 had it both there and here; 3 first got it here.' These figures speak very plainly for themselves."

In America we are indebted to the indefatigable labors of Dr. Charles Dennison, of Colorado, far more than to any other individual, for his masterly studies of the Rocky Mountain region as health resores.

Granting that consumption is dependent upon the development of a bacillus within the lung, what may we legitimately expect from climate for its destruction or expulsion? Its propagation, so far as we now know, is dependent upon a proper soil, which means the furnishing of suitable nutriment in the form of albuminoids; a continuous temperature and a proper amount of moisture. These supplied, the bacilli reproduce abundantly externally to the individual, and that they do not in any manner lose their pathogenic qualities is shown by their ready reproduction and development, following inoculation, causing the death of healthy animals.

Do climatic changes modify these factors? Much has been claimed for and written upon the advantages of a dry climate in consumption. There are other reasons why this may give benefit, but I suppose no one will contend that the fluids in the tissues of the lungs are materially lessoned thereby, or that while life continues, these can by any manner of means be so diminished as to prevent bacillary development. The heat point for the bacillary growth must be maintained so long as the individual exists, for its reduction to a point to interfere with its development must be incompatible with the conditions possible for the maintenance of the life of the individual.

Given the depreciation of the vitality of the tissues locally, and that the albuminoids requisite to furnish food for the unwelcome tenants continue, and we safely infer, from what we now know, that an aseptic atmosphere no matter how dry, or rarefied, cannot alone furnish the factors of cure.

The *role* of bacterial reproduction varies greatly in certain diseases. Thus in scarlet fever, measles, small-pox, once having run its course the bacterial development is at an end, and only in the most exceptional of conditions can be reproduced in the same individual. In a measure, this is true of typhoid, typhus and yellow fever, also diphtheria, and perhaps other diseases. Remittent or malarial fever is an exception, and yet, after a time, the individual either takes on an increased resisting power to the disease, or the conditions necessary for its development are changed and so-called acclimatisation follows. However, the residents of a malarious country never entirely escape its influence. That it is a new infection, rather than the development of the original seed, appears probable, since ordinarily a residence in a country free from malaria soon causes the disease to disappear While thus much may be determined from experience, and the sufferer from this disease promised exemption by change of climate, sufficient is not yet known of the *role* of the bacillus malaria from which to deduce more that the most general conclusions.

Enough is known of the bacillus tuberculosis to show that it is found in all countries, making its greatest inroads in thickly settled communities and is most prevalent in cold, damp localities. This latter condition may be considered favorable since, thereby, catarrhal conditions of the respiratory passages are induced and thus a soil is prepared favorable for its develovment.

For similar reasons the general vitality, or resisting power of the individual is reduced. That the person thus subject to catarrhal inflammations should be greatly benefitted by breathing an aseptic air is easily conceived, since such an individual residing in a city can scarcely escape the breathing of an in-infectious atmosphere.

Every pathologist knows that a considerably greater percentage of cures in consumption is effected than is generally supposed. It is not rare to find localized, cicatricial and calcareous degeneration in the apices of lungs, otherwise healthy, which may be traced to disease incurred even in early life. If caused by bacilli, why did these cases recover? The answer must be that the conditions for the proper development of the microbe did not pertain; that in some way the vital, resisting power of the tissues was superior to the invasion of the disease; that cells were proliferated as a protective wall, so to speak, against the invasion of the microbe which did not furnish the albuminous food necessary for its growth. Thus shut in, the disease is necessarily limited, and bacterial development ceases although the spores may indefinitely retain their vitality.

See, in his recent treatise upon "The Bacillary Phthisis," discusses, at a considerable length, the question of mycrophytic life, especially that of the bacillus tuberculosis in high altitudes and considers such a climate, per consequent, of prophylactic character.

Recent very interesting experiments conducted in the pure air of the Adirondack region show that the development of the bacillus in animals following inoculation and infection does not in any wise differ from the labratory experiments carried on in the large cities.

Dr. Harrold Williams, in a carefully prepared paper read before the Massachusetts Medical Society the present year, reviews certain phases of the question of the adaptability of climate to individuals, and in summing up says: "It seems that we must admit, in the present state of our knowledge, the meteorologic differences of climate have been proved to be of little importance in the treatment of phthisis, and furthermore that clinical evidence would support this conclusion, for the burden of proof lies with those advocates who plead in favor of special climates, and such proof it seems to me is yet to be forthcoming."

This is more than I am willing to admit, since we should look for aid to the infected individual from climatic change, not in the breathing of an "anti-bacillary atmosphere," but in the placing of the patient in surroundings suitable, if possible, to strengthen his weakened vital, resisting power, and rein force his cellular tissue, so that it may be superior to the attack of the would-be destroyer, and although we do not know all the conditions requisite for counteracting the growth of morbific bacilli, we are assured that healthy tissues and blood imperfectly furnish the required pabulum. Were it only necessary to breath an anti-bacillary atmosphere, science could solve the problem and give to the sick chamber an antiseptic air fatal to bacterial growth. Many have builded hopes upon such remedial agents and, perhaps, in a certain measure correctly, but given a "pneumatic cabinet" and antiseptic inhalations, we should not expect to affect a diseased gland, a caseous nodule, only, in a certain limited degree, a cavity even, since the air inspired goes almost wholly into the less diseased and healthy portions.

When our present knowledge of infectious diseases is thus reviewed, are we to ignore, as some recent writers have done, the entire question of benefit to be obtained from climatic change? On the contrary, it appears therefrom that we have sufficient data by which to add, emphasize and encourage climatic investigations and confirm previous experience, even if the theories, under which it was sought, do not prove true.

Equability and dryness of the air are certainly important, well-recognized factors in the alleviation of the respiratory tract, and this alone is sufficient to make these conditions of value in the selection of a residence for such invalids. Atmospheric moisture, in cloud or vapor, is detrimental by lessening the sunlight; and the clear skies of any land are justly prized. This is sometimes called the diathermacy of the atmosphere and this is increased by the rarefaction of the air. Nearly every one has observed the intensified effect of the sun's rays at high altitudes.

Clinical observations teaches that, in the invalid able to endure it, active out-of-doors exercise, in elevated localities, has an especial invigorating effect upon the respiratory function and apparatus; the circulation is improved, thereby increasing the oxydation of the tissucs, as well as producing a better cellular nutrition and elimination of the effete material. The lowering of the atmospheric pressure also tends to diminish the circulation of the deeper tissues, which in certain conditions of the organs is of great importance, as for example, in renal, heptic, or cerebral congestions, while on the other hand, the blood-flow to the surfaces is greatly augmented, an important factor in conditions of defective or perverted cutaneous nutrition. The effect of this is shown in increased appetite and improved digestion, until the imperfectly prepared food, too often forced by necessity to supply the demand, is taken with a relish never known at home and digested without knowledge of organs or process.

With a labor only known to those who have worked in such direction, Dr. Dennison has utilized the mass of statistics gathered by the Signal Service Department of our Government and placed before the profession his Seasonal Maps of the United States. These are worthy the careful study of every physician and teach many facts of the greatest importance. Under date of June, 1885, Dr. D. writes in regard to Colorado: "In my practice of twelve years in Colorado, it has been very seldom that I have known of the origination of phthisis, chronic pneumonia, etc. I do not remember to have written a certificate of death for one uncomplicated case of phthisis, originating in Colorado. Of course there are such cases. I have one under my care at the present time. As civilization progresses here, it will not be strange if the disease becomes much more frequently met with in thickly settled districts, made up as they are of a considerable scattering of regenerated invalids. The weight of evidence is so decidedly in favor of elevation as an important factor in the climate for the consumptive, that it will not answer for anyone who has not personally investigated the Rocky Mountain regions, or similar elevated resorts, to say that nothing has been proved for elevation. Everything has been proved, since all the most desirable attributes, dryness, coolness, electricity, sandy soil, perfect drainage, and last, but not least, *purity of atmosphere* are found and increased with elevtion above the sea-level."

Dr. H. Weber, in his excelent resume of the subject of climatic treatment of phthisis given in the Croonian lectures, published in the *British Medical Journal* the present year, emphasises the advantage gained by the invalid not only from elevation, but also by the cold dry air, and makes the distinction that in the sunshine the coldness pertains rather to the atmosphere than to the individual. Speaking of his personal experience in the high Alps in November at the elevation of 10,000 feet, without an overcoat, he says: "As long as I remained in the sunshine I never felt warmer in August or September in the same localities; but in the shade an overcoat would have been just a comfort, though I did not feel cold, in spite of the temperature of only 20 to 25 degrees F., the air being perfectly calm. A black bulb thermometer *in vacuo* showed in the sun between 80 and 92 degrees F., while an ordinary thermometer registered only between 31 and 32 degrees F." He sums up the advantages attained in mountain health resorts to be as follows: "1st, the atmospheric purity or aseptic nature, the comparative absence of floating matter. 2nd, dryness of the air and soil, comparative absence of mist. 3rd, the coolness of the air temperature and the great warmth of the sun temperature. 4th, the rarefaction and low pressure of the air. 5th, the intensity of the light. 6th, the stillness of the air in winter. 7th, a large amount of ozone. The effects on the invalid suited to such climates are, increase of appetite, improvement of sanguinification and general nutrition, strengthening of the heart and circulation, raising of muscular and nervous energy and of activity of the skin."

Observations have been made in some of the South American high altitude stations, as at Junja of more than 10,000 feet above the sea. The range of temperature during an entire year was between 50 and 60 degrees F., with a sky always clear and sunny and atmosphere pure and bracing which invites to out-of-doors exercise and enjoyment. In an atmosphere, as cold as the winter in the Alps or Rocky Mountains, it is essential to select localities where protection from wind is attained, since the motion of an atmosphere thus cold, abstracts heat rapidly. In broad stretches of equable cold, the winds are reduced to a minimum and the absence of moisture greatly increases the hours of sunshine. The chief objection to the Rocky Mountain health resorts lies in the distance from the centers of population. The White mountain region affords a very considerable elevation, but, judged in the light of the above experience, too little to secure much good from the rarefaction of the air, since Bethlehem is the higher location of the villages and this is only eighteen hundred feet above the sea-level. The Adriondacks are of less elevation, but possess the advantage over the White mountain in being sparsely inhabited, an almost unbroken forest, an elevated plateau furnishing an equable, cool summer and a decidedly cold winter climate. Through the influence of Dr. Loomis, of New York, a sanitarium has been established for patients to remain the entire year, and good results have been attained, but it is difficult of access and offers very little advantage from rarefaction of the atmosphere.

The mountains of the Allegany range have been known to some extent as affording interesting and health-giving resorts for summer recreation, and in the southern ranges furnishing summer homes for the residents of the Southern States. The entire section is free from malaria, and Ashville in western North Carolina has grown to a village of about five thousand inhabitants, chiefly as a summer resort.

